# Disrupted Care Continuity: Testing Associations between Social Networks and Transition Success for Children with Autism

**DOI:** 10.3390/socsci10070247

**Published:** 2021-06-28

**Authors:** Elizabeth McGhee Hassrick, Wendy Shih, Heather J. Nuske, Sarah F. Vejnoska, Samantha Hochheimer, Deborah E. Linares, Jonas Ventimiglia, Kathleen Carley, Aubyn C. Stahmer, Tristram Smith, David Mandell, Connie Kasari

**Affiliations:** 1 A.J. Drexel Autism Institute, Drexel University, Philadelphia, PA 19104, USA;; 2 Center for Autism Research & Treatment, Department of Psychiatry, UCLA Semel Institute, Los Angeles Graduate School of Education, Information Studies, University of California, Los Angeles, CA 90024, USA;; 3 Center for Mental Health Policy and Services Research, Perelman School of Medicine, University of Pennsylvania, Philadelphia, PA 19104, USA;; 4 Departments of Psychiatry, Psychology & Human Development, Davis MIND Institute, University of California, Sacramento, CA 95817, USA;; 5 Strong Center for Developmental Disabilities, Division of Developmental and Behavioral Pediatrics, University of Rochester Medical Center, Rochester, NY 14642, USA;; 6 National Institute on Minority Health and Health Disparities, National Institutes of Health, Bethesda, MD 20817, USA;; 7 School of Computer Science at the Institute for Software Research, Carnegie Mellon University, Pittsburgh, PA 15213, USA;

**Keywords:** continuity of care, social networks, autism, school transitions, parent engagement, lower income families

## Abstract

Children with autism situated in lower income families often receive intensive educational interventions as their primary form of treatment, due to financial barriers for community interventions. However, the continuity of care can be disrupted by school transitions. The quality of social relationships during the transition to a new school among parents, school staff and community providers, called the team-around-the-child (TAC), can potentially buffer a child with autism from the adverse effects caused by care disruptions. Qualities of social relationships, including trust and collaborative problem solving, can be measured using social network analysis. This study investigates if two different types of TAC relationships, defined as (1) the level of trust among team members and (2) the degree of collaborative problem solving among team members, are associated with perceived successful transitions for children with autism from lower income families. Findings suggested that TAC trust is significantly associated with the outcome of transition success for children with autism immediately post-transition.

## 1. Introduction

Autism is a serious disorder affecting perhaps 1 in 54 children in the United States (Maenner et al. 2020). Intervention in childhood can have a significant effect on development. Children with autism from lower-income families often rely on intensive educational interventions as their primary form of treatment (Lord et al. 2018; Lord and McGee 2001). For children with autism, these interventions often require collaboration between the child’s home, school and community healthcare providers, sometimes referred to as the team-around-the-child (TAC) (Limbrick 2007; McGhee Hassrick 2019), as many children with autism have co-occurring disorders and can experience difficulty transferring skills learned in one setting to another.

However, during periods of educational disruption, such as a transition to a new school, the TAC can experience high turnover, when key members of the clinical support team from the pre-transition school context exit and new members in the new post transition context begin, potentially disrupting the continuity of care and negatively impacting transition success for the child. Times of school transition can create disruption for the TAC, as well as for the child. At the end of the school year, immediately before transition begins, the TAC faces challenges simultaneously ending clinical and educational supports in the current school, while attempting to prepare the child and their family for an unknown new clinical team at a future school. Similarly, during the first few weeks at the new school, the child’s new TAC must strive to quickly determine and implement the needed interventions to assist the children with autism to acclimatize to new providers and learn new routines, all while the child’s family attempts to forge new relationships during a time of disruption and stress.

Prior work with general education students measuring the impact of school transition produced mixed results, with some studies predicting adverse impact on outcomes due to disruptions during school transitions (Roderick and Camburn 1999; Ruble and Seidman 1996; Simmons and Blyth 1987) and other studies suggesting no negative impact, with a small apparent benefit for isolated youth who experienced a “fresh start” at their new school (Weiss and Bearman 2007). However, no prior social network studies have examined the quality of social relationships for the TAC immediately before and immediately after key developmental school transitions for children with autism. Additionally, no studies have investigated the transition experiences of children with autism from lower income families who receive the majority of their interventions in educational contexts. The aim of this study is to test the association between two qualities of social connections among the team-around-the-child (TAC), including (1) trust among TAC members and (2) problem-solving engagement among TAC members, with transition outcomes for children with autism from lower income families.

### 1.1. Unequal Continuity of Care during School Transitions

Inequality in access to social capital could adversely impact the ability of the TAC to provide continuity of care for children with autism from lower-resource families during disruptive schooling transitions. A smooth, well-orchestrated transition for a child with autism to a new school requires many details, big and small, that are challenging to coordinate and accomplish. For example, timely placement several months before transition would provide pre-transition teachers with actionable knowledge about how to prepare their student for the specific expectations and contextual demands of their new school. Working relationships between pre and post providers can support the development of a well-informed transition plan. Economic disparities can adversely impact the amount and quality of support and resources embedded in social networks (Bourdieu 1977; Lareau 1989, 2011, 2015; Lin 2017; Small 2009). Parents of children with disabilities face additional challenges, navigating educational systems of services and supports (Burke and Goldman 2015; Trainor 2010; Kalyanpur et al. 2000; Ong-Dean 2009), increasing the need for access to social capital that could support educational disruptions (McGhee Hassrick 2019; Nuske et al. 2019).

Larger urban districts with fewer resources that serve a majority of lower income students often face challenges securing information in advance about the placement for children with autism. Placement information can be delayed until the summer after pre-transition schools have ended or, in some cases, after the beginning of the new school year has begun (Darling-Hammond et al. 2005; Neild 2009). Districts with less resources struggle to provide staff with the necessary resources for the TAC to effectively collaborate, for example paid release time to attend transition meetings or paid coordination time to create a “warm hand off” between pre and post transition staff. Such uncertain conditions make preparation for educational transition difficult.

Post-transition also poses its own set of challenges, such as the need to establish a new multi-system team, where home, school and community providers must quickly establish working relationships to help a child with autism smoothly transition to new school settings. While the TAC can build trust and come together to solve problems associated with transition to support care continuity, such teaming efforts can be particularly challenging to establish as children and their parents experience anxiety and stress anticipating the upcoming disruption, or when the new school team has little information about the child and their unique needs. Transition contexts that result in high levels of uncertainly can disrupt established relationships and the formation of new relationships for the TAC.

The quality of social connections among parents, school staff and community providers immediately pre- or post-transition to a new school can potentially buffer a child with autism from the adverse effects of continuity of care disruptions. Trust and collaborative problem solving can serve as social resources for the TAC, helping TAC members support the child to prepare for the transition to a new school setting and adjust to the new school setting once they have completed transition. Social resources, often referred to as social capital (Coleman 1990), can provide advantages for the TAC of children with autism, such as timely information sharing among the TAC about how to best maintain continuity of care during new school transitions. Supportive social relationships are important resources during big life changes for children, especially children with autism who experience difficulties with social communication, peer connections, resistance to change (Cuccaro et al. 2003) and intolerance for uncertainty (Boulter et al. 2014). Children with autism experience challenges during major school transitions, including difficulties managing anxiety, communicating with classmates and school staff members, and adjusting to new routines (Nuske et al. 2019; Stoner et al. 2007).

### 1.2. Team-around-the-Child: Trust and Transition Success

Affective relationships, such as trust, suggest shared norms, values and expectations that can serve as a stabilizing force during times of duress and change (Coleman 1990). Shared norms and values suggest alignment of goals and protection from shared vulnerabilities during difficult time periods (Bryk and Schneider 2003). People who share similar attributes, cultures, contexts, or roles, are more likely to experience homophily (Lazarsfeld and Merton 1954), which can engender trust. Research on trust between different groups, for example, family members and school staff, is limited, in part because cross-group trust is difficult to operationalize (Adams and Christenson 2000). Literature on trust between families of children with autism and school staff is particularly scarce; however, the literature that does exist suggests that home–school communication, collaboration, and exchange of information during school transitions is essential to establish trust between parents and school staff (Adams and Christenson 2000; Fontil et al. 2019; Schischka et al. 2012; Starr et al. 2016; Rous et al. 2007).

Relationship building, including building trust and rapport between schools and parents, is essential for culturally and linguistically diverse families (Starr et al. 2016). For children with autism, trust between families and schools is related to shared expectations about their unique needs and knowledge about the characteristics of autism. However, educators that lack knowledge and training about autism may experience difficulties understanding autistic students, their behavior, and their needs. This can strain relationships between teachers and parents, resulting in a lack of trust (Starr et al. 2016; Fontil et al. 2019; Richter et al. 2019). Broken trust between families and schools can lead to decreased communication, refusal by parents to accept suggestions from school personnel, and lowered expectations by the parent of positive outcomes for their child (Lake and Billingsley 2000).

### 1.3. Team-around-the-Child: Problem Solving and Transition Success

Each member of a child’s TAC faces different opportunities and challenges solving problems and sharing solutions with one another. Examples of problem solving include sharing advice about instructional tips among teachers who teach the same child, expertise sharing between community professionals and school providers about a specific treatment implemented at school and in the community, or problem solving between parents and school staff about how to co-implement a behavioral plan that is consistent across home and school.

Team problem solving is especially important in children with autism who present with more challenges. Many children with autism have problem behaviors, such as aggression, self-injury, and elopement (Baumeister et al. 2016; Brereton et al. 2006; Gray et al. 2014; Matson and Cervantes 2014). As the function of problem behaviors, as well as the strategies to mitigate them, are context dependent (Steinbrenner et al. 2020), children with problem behavior may require more planning and coordination across and within teams to ensure that problem behaviors are not increased in the post-transition setting. For example, for a child whose problem behavior serves the function of the communication of his/her needs, individualized communication supports would need to be in place in the post-transition period to ensure their problem behavior does not increase in that setting. Given the stress and anxiety often experienced by children with autism during sensitive periods of change, such as a new school transition, difficulty adjusting to the new routines and physical environment (or anticipating these upcoming changes), may also trigger new challenging behaviors in a child before and/or after the transition (e.g., elopement to serve a stress-avoidance function. Indeed, our review on school transitions in children with autism identified that an increase in challenging behavior was a common difficulty in children with autism during school transitions (Nuske et al. 2019).

Instrumental support among school staff members affects teacher performance. Several studies suggest that teachers seek out advice and support from their fellow teachers to help them achieve their instructional goals (Bidwell 2001; Coburn et al. 2010; Penuel et al. 2009). When teachers turn to their same grade or same topic colleagues to seek out advice about how to address problems that emerge in their classrooms, they create informal local problem-solving systems that provide resources to improve their everyday work in the classroom with their students (Rowan 1990; Smylie 1997).

Teachers of students with disabilities who participate in schools where school staff engage as “co-learners” and seek one another out for iterative problem-solving activities, potentially have access to valuable resources that help them address challenges in the students’ everyday learning (Zaretsky 2005). Frequent communication between school administration, social workers and teachers can support individualized instruction in the classroom (Hassrick et al. 2017). While advice seeking and problem-solving engagement represent important resources among school staff, little research has investigated how teachers and other school staff engage one another to support children with autism during potentially disruptive school transitions.

Federal mandates regarding support for students with disabilities (Individuals with Disabilities Education Act, IDEA) and best practice suggest that coordination and collaboration by the entire home, school and community healthcare team members impact child outcomes. While some studies have tested associations among dyads that support children with autism (e.g., teacher × teacher; teacher × administrator; teacher × parent dyads), no studies have investigated how the TAC might impact care continuity for children with autism before key school transitions and after key school transitions (Azad et al. 2016; Quintero and McIntyre 2011; Nuske et al. 2019). Although there are a few current studies, there is an absence of new research on this topic. To answer this gap in the literature, we used social network data to test the hypothesis that the quality of TAC relationships shapes transition success for children with autism.

## 2. Methods

### 2.1. Community-Based Research Partnership

This study was part of a large US-based multi-site, multidisciplinary, collaborative research network focused on advancing the evidence base for effective behavioral interventions to improve health and well-being in lower-resourced families of children with autism. Local Institutional Review Boards at each recruiting site approved study procedures and utilized reliance agreements. The pilot data for this study were collected at public schools in three different states to inform later intervention development for caregivers of children with autism who were experiencing school transitions. To ensure that all study design, protocols, recruitment, and implementation considered the needs of lower income families with children with autism, a community-based research partnership was established at all study recruitment sites. Community partners were identified based on their role and involvement with school-based transition processes for lower income families with children on the autism spectrum. A wide range of professionals were engaged, including school district administration, early intervention system administration and providers, teachers, specialized service providers, and agency representatives. At some sites, partnerships also included community organizations focused on broader child, family, and community-wide initiatives. To ensure the representation of the target population, caregivers of children with autism were recruited to each partnership. Partnership meetings occurred monthly for the early stages of the project and continued throughout the recruitment phase. Meetings were facilitated by a member of the research team. Project updates were provided, and then conversation was facilitated related to study process, such as review of materials in development, sharing recruitment ideas, and troubleshooting barriers. Each meeting lasted approximately an hour, and participants were compensated with a USD 25 gift card for their time. Collective partnership feedback was shared across sites on a bi-monthly call. All study design, protocols, recruitment, and implementation for this study were collaboratively produced by the community research partnership.

### 2.2. Study Participants

Participants were recruited from participating public schools in three different states. There is substantial variability in the transition services provided for students with ASD across schools in the US, with schools with more supportive transition practices offering transition planning meetings, identifying key personnel to facilitate the transition, and communicating often with parents and students about the transition (Nuske et al. 2019). The variability in provided services is often related to school characteristics, resources, and administrative support (Nuske et al. 2019). Participants included key home, school, and healthcare providers, each of whom provided intervention support for children with autism enrolled in the study during the child’s pre-transition period (*n* = 8 children, including 2 children experiencing a transition to kindergarten and 6 children experiencing a transition to middle or high school) or the child’s post-transition period (*n* = 7 children, including 2 children experiencing a transition to kindergarten and 6 children experiencing a transition to middle or high school). To be eligible for the study, children had an autism diagnosis in their individual education program (IEP), were from a low resourced family (defined as having a household income that was 200% at or above the federal poverty threshold, adjusted for family size) and were experiencing a transition to a new school during a key developmental period; either from prekindergarten to kindergarten or elementary to middle school or high school. Federal poverty levels were used to provide a standard threshold across the three states. Data were collected during each child’s pre-transition period, six weeks before the end of the school year, and during each child’s post-transition enrollment period, three months post entry into the new school.

We used a snowball process to identify key home, school, and community providers for each child to establish the boundary for the network analysis. First, using the Hierarchical Mapping Technique (HMT) (Antonucci 1986), caregivers were asked to identify the most important people who helped them help their child. These names and their contact information was listed in the inner circle of the HMT diagram. Next, caregivers identified other people who were important, but not as important as those in the inner circle. These were listed in the next circle. Caregivers then identified up to five key providers from the HMT. With caregiver permission, key providers were recruited for participation in the study. Key providers identified by the caregiver also completed the HMT, as the second wave of the snowball. Their 5 key providers, if not already listed by the caregivers, were also contacted and were asked to complete the HTM. This second set of key providers also identified up to 5 key providers, and if not already listed by the caregivers or the first round of key providers, were also contacted and were asked to complete the HTM. The snowball process ended after the third round and no more key providers were recruited. Child teams, where greater than 50% of key providers, identified through the snowball process, agreed to participate in the study were included in the analysis, yielding 8 pre-transition teams and 7 post transition teams.

Due to sample size and high turnover during pre- and post-transition among the child’s TAC, we conducted cross-sectional analyses for the pre- and post-time points. For the pre-transition cross-sectional analysis, 44 TAC members participated in the study (*n* = 44 overall; *n* = 13 home providers; *n* = 31 school and community healthcare providers). See Table 1. For the post-transition cross-sectional analysis, 48 TAC members were identified (*n* = 48 overall; *n* = 13 home providers; *n* = 35 school and community healthcare providers); see Table 2.

### 2.3. Study Variables

Several types of variables were used in analyses, including transition success as the outcome, social network variables as the main predictors and covariates for role and child behavior.

#### 2.3.1. Team-around-the-Child Rated Transition Success as Outcome Variable

The primary outcome was TAC ratings of perceived transition success at two different time points; once before the child transitioned to their new school (pre-transition) and once after the child transitioned to their new school (post-transition). The Transition Evaluation Questionnaire (TEQ), a transition success measure, was developed for the current study to assess TAC perceptions of the student’s transition success. Key members of the child’s TAC answered the following question. “How successful is the child’s transition process to their new school?” The scale was as follows: 1 (not successful); 2; 3 (somewhat successful); 4; 5 (very successful). The present study utilized a binary scale in which ratings of 1–3 are considered low transition success and ratings of 4–5 are considered high transition success. The TEQ was collected from the child’s TAC at pre-transition (last 6 weeks of school year before transition), and again at post-transition, three months into new school placement.

#### 2.3.2. Social Network Variables as Main Predictors

Standard social network measures for indegree and outdegree (Wasserman and Faust 1994) were used to characterize the degree of trust and problem solving for each person in the TAC (See Table 3). To measure trust, we asked each team member to identify trusted advisors on the child’s team. The participant identified other team members who “gave them good ideas” about how to help the child with autism. Trust out degree was measured by the number of people on the team who were trusted by participant/team size. Trust in degree was measured by the # of people on the team who trusted the participant/team size. Total Trust was the sum of Trust in degree + Trust out degree. Problem solving out-degree was measured by the # of people on the team who the participant sought out to solve problems for the child/team size. Problem solving in-degree was measured by the # of people on the team who sought out the participant to solve problems for the child/team size. Total Problem solving was measured by the sum of Problem solving in degree + Problem solving out degree.

#### 2.3.3. Roles as Covariates

The team-around-the-child included parents, family members, are characterized in the analysis as the “Home Team” and school providers, including special education teachers (i.e., general education teachers, school clinicians and aides), and healthcare providers (i.e., community behavioral therapists, doctors) are characterized as the “Provider Team”.

#### 2.3.4. Child Problem Behavior as Covariate

Problem behaviors in children with autism can potentially make school transitions more challenging. As a control variable, all TAC members completed a 12-point behavioral concerns questionnaire, adapted from Quintero and McIntyre (2011), about the target child with autism. To measure each participant’s level of concern about the target child’s behavior, each participant answered the following question. “Please tell us how much each of the following areas concerns you, right now, about the transitioning child.” The scale was as follows: 1 = no concerns; 2 = a few; 3 = some; 4 = many concerns.

### 2.4. Data Analysis

We conducted two cross-sectional analyses using logistic regression models. First, we analyzed data collected during the pre-transition period and second, we analyzed data from the post-transition period.

## 3. Results

We found that TAC members immediately post-transition, with more trusting TAC connections, were significantly more likely to rate the child’s post-transition as successful. Problem solving ties were marginally associated with transition success in the post-transition period. However, none of the social network ties were associated with future transition success during the pre-transition period.

### Associations between Social Networks and Transition Success

Associations between social network characteristics and successful transition are reported below for the first analysis of the pre-transition period (Table 4) and the second analysis for the post-transition period (Table 5). For the pre-transition time period, TAC members predict their perception of how the child’s transition will go. In post-transition time period analysis, the TAC members rated the transition experience of the child post-transition to their new school. For the cross-sectional analyses, marginally significant results are reported where *p* < 0.10, as well as significantly associated results where *p* < 0.05.

TAC members in the post-transition period who trusted more of their fellow TAC members were 9.44 times more likely to rate the child’s transition as successful (significant with *p* < 0.05). TAC members in post-transition who engaged in problem solving for the child with more of their fellow TAC members were 7.58 times more likely to rate the child’s transition as successful in the post-transition period (marginally significant with *p* < 0.10).

However, during the pre-transition period, none of the social network ties were significantly associated with TAC members’ ratings of a success transition for the child during the pre-transition period and both were negatively associated with perceived transition success. TAC providers at school and healthcare settings were between 4.41 and 5.39 times more likely to rate that the child would have a successful transition than home providers during the pre-transition period (marginally significant with *p* < 0.10) and between 19.52 and 15.24 times more likely to rate the child’s transition as successful during the post-transition period (significant with *p* < 0.05).

TAC members who rated the child as having more severe behavioral issues were approximately 50% less likely to rate that the child would experience transition success during the pre-transition period (significant with *p* < 0.05). In the post-transition period, TAC members who rated the child as having more severe behavioral issues were between 68% and 73% less likely to rate that the child has experienced a transition success during the post-transition period (significant with *p* < 0.001).

## 4. Discussion

We found that TAC members with high trust for other TAC members were more likely to rate the child’s post transition as successful, which is consistent with previous work in this area (Matson and Nebel-Schwalm 2007). This finding suggests the implementation of teacher strategies for establishing trusting relationships among the child’s TAC members to support a successful school transition experience for autistic children (Nuske et al. 2019). Interestingly, this finding was specific to post-transition teams, suggesting that the development of trust is particularly critical in new school TAC. Though the causal direction cannot be delineated from these findings, and both directions are plausible (team trust promoting a successful transition vs. a successful transition promoting team trust), these findings confirm the importance of team trust for new school transitions.

Problem solving with team members was associated with trust within teams. Given that trust takes time to develop, and this finding was consistent across pre- and post-transition teams, one may posit that the directionality, such that people trusted the people with whom they engaged in problem solving. Active collaboration among team members promotes trust within teams, and is consistent with findings showing that home-school communication, collaboration, and exchange of information enhances trust within teams during school transitions (Adams and Christenson 2000; Fontil et al. 2019; Rous and Hallam 2012; Schischka et al. 2012; Starr et al. 2016). However, these findings need confirmation in future research using longitudinal samples with multiple data points of the post-transition teams.

We found that children’s problem behaviors were associated with less perceived transition success during both pre- and post-transition. This result extends the evidence, showing that problem behaviors negatively affect other areas of development, such as opportunities for socialization and learning (Matson and Goldin 2013) (Matson and Nebel-Schwalm 2007) and emphasize the need to address problem behaviors in order to ensure a smooth new school transition for children with autism.

The findings suggested that providers were more likely to perceive transition success than families during the pre- and post-transition periods. The source of this discrepancy was beyond the scope of the current study and should be followed up in future research. However, given that parents often feel worried, alienated, discontent and powerless during the new school transition period (Nuske et al. 2019), it is understandable that their outlook on their child’s transition might not be like the perceived ratings for professionals. This result further suggests parent’s involvement in the school transition process is a worthy target of intervention.

### 4.1. Clinical Implications

This study provides insight about how team dynamics shape success for autistic children from lower resourced families in public school settings. The findings of the current study suggest that promoting trust within teams, as well as engaging families in problem solving and addressing any problem behaviors should all be included in interventions designed to promote successful school transitions in children with autism. Intervention development for new school transitions in children with autism is still in its infancy. One transition intervention, the Systemic Transition in Education Program for Autism Spectrum Disorder (STEP-ASD) (Mandy et al. 2016) targets students with autism who are transitioning to mainstream (i.e., regular education) secondary schools, and while it has not yet been rigorously tested, it does target shared problem solving between teachers and parents, and addresses students’ behavioral needs. No transition support interventions have been developed that address the specific continuity of care needs of children with autism from lower resourced families.

### 4.2. Limitations

This study is not without limitations. First, despite the novel methods employed in this study to examine the association of social network characteristics on transition success, the study was not designed to examine causal relations. Second, the small number of participants impacts the conclusions from this study in two unique ways by limiting the ability to generalize these findings and the non-significant results could be due to the covariates having small effect sizes, and that the study did not have the power to detect. Third, investigating school transitions presents methodological challenges due to team attrition and post-transition additions, as team members often do not stay constant throughout the transition. Nevertheless, future work should use a longitudinal design with a robust enough sample to investigate the impact of team characteristics on new school transition success using a longitudinal design involving pre-transition school team members at post-transition follow-up, or by following up with post-transition teams after their initial measure timepoint.

## 5. Conclusions

The current study found that engaging families in problem solving, promoting trust within teams and addressing any child problem behaviors promotes successful school transitions for children with autism during the post-transition period. These findings highlight the importance of providing the Team-around-the-Child with support and resources to build trust to address the complex needs of children with autism during times of major school transitions.

## Figures and Tables

**Table 1.** Pre-transition participant sample.

Pre-Transition Sample Characteristics	*N*	Min	Max	Freq/M	%/SD
Child Attributes (*n* = 8)					
Male: *n* (%)	8			7	87%
Child Behavior (mean)	8	1.57	3.50	2.56	0.67
Child Transition Success	8	2.50	3.75	3.17	0.37
Child Transition Type					
Primary Transition	8	0.00	1.00	0.25	0.46
Secondary Transition	8	0.00	1.00	0.75	0.46
Family Income Level					
Income under $35,000	8	0.00	1.00	0.75	0.46
Income $35,000–$45,000	8	0.00	1.00	0.25	0.46
Home Attributes (*n* = 13)					
College Degree or Higher	10	0.00	1.00	0.30	0.48
Gender (Female)	13	0.00	1.00	0.77	0.44
Problem Behavior	13	1.00	4.00	2.69	0.85
Provider Attributes (*n* = 31)					
College Degree or Higher	25	0.00	1.00	0.96	0.20
Gender (Female)	31	0.00	1.00	0.77	0.43
Problem Behavior	31	1.00	4.00	2.45	1.03
Autism Experience	31	0.00	1.00	0.87	0.34
Years in current role	31	0.00	31.00	9.27	8.61

**Table 2.** Post-transition participant sample.

Post-Transition Sample Characteristics	*N*	Min	Max	Freq/M	%/SD
Child Attributes (*n* = 7)					
Male: *n* (%)	7			7	100%
Child Challenging Behavior	7	1.67	3.54	2.49	0.70
Child Transition Success	7	2.33	4.50	3.58	0.94
Child Transition Type					
Primary Transition	7	0.00	1.00	0.29	0.49
Secondary Transition	7	0.00	1.00	0.71	0.49
Family Income Level: *n* (%)					
Income under $35,000	7			7	100%
Home Team Attributes (*n* = 13)					
College Degree or Higher	10	0.00	1.00	0.30	0.48
Gender (Female)	13	0.00	1.00	0.77	0.44
Child Behavior	13	1.00	4.00	2.54	1.39
Provider Team Attributes (*n* = 35)					
College Degree or Higher	32	0.00	1.00	0.94	0.25
Female	33	0.00	1.00	0.73	0.45
Child Behavior	35	1.00	4.00	2.80	0.96
Autism Experience	32	0.00	1.00	0.59	0.50
Years in current role	35	0.00	23.00	5.12	6.79

**Table 3.** Definitions of social network variables.

Network Variables	Description
***Team Affective Engagement***	
Trust Out Degree	Number of people on the team who were trusted by participant/team size.
Trust In Degree	Number of people on the team who trusted the participant/team size.
Total Trust	Sum of Trust Indegree + Outdegree
***Team Instrumental Engagement***	
Problem Solving Out Degree	Number of people on the team who were sought out for problem solving/team size.
Problem Solving In Degree	Number of people who sought out the participant for problem solving/team size.
Total Problem Solving	Sum of Problem Solving Indegree + Outdegree

**Table 4.** Pre-transition network predictors of transition success.

	Child Transition Success Pre-Transition Time Period								
	Model 1			Model 2			Model 3		
	B	SE	Odds	B	SE	Odds	B	SE	Odds
Intercept	0.61	1.12	1.84	0.68	1.15	1.98	0.76	1.13	2.13
**Covariates**									
Provider	1.48*	0.78	4.41	1.56*	0.84	4.77	1.68*	0.83	5.39
Child Behavior	−0.70 **	0.36	0.5	−0.69 *	0.36	0.5	−0.61	0.37	0.54
**Network Variables**									
Team Trust Overall				−0.28	1.02	0.76			
Team PS Overall							−0.89	0.94	0.41

** *p* < 0.05,

* *p* < 0.10.

**Table 5.** Post-transition network predictors of transition success.

	Chiild Transition Success Post-Transition Time Period								
	Model 1			Model 2			Model 3		
	B	SE	Odds	B	SE	Odds	B	SE	Odds
Intercept	1.94*	1.07	6.96	0.55	1.22	1.73	0.87	1.19	2.38
**Covariates**									
Provider	2.97 ***	1.12	19.52	2.53 **	1.1	12.59	2.72 ***	1.09	15.24
Child Behavior	−1.32 ****	0.46	0.27	−1.15 ***	0.43	0.32	−1.29 ****	0.43	0.27
**Network Variables**									
Team Trust Overall				2.25 **	1.04	9.44			
Team PS Overall							2.03*	1.06	7.58

**** *p* < 0.001,

*** *p* < 0.01,

** *p* < 0.05,

* *p* < 0.10.
